# Insurance companies’ lack of LGBTQ+ affirmation: discrimination, distrust, and dissatisfaction among LGBTQ+ people

**DOI:** 10.3389/fsoc.2025.1569519

**Published:** 2025-07-18

**Authors:** Dustin Z. Nowaskie, Dehandra Blackwood, Frank Garcia, Jorge D. Flautero

**Affiliations:** ^1^Department of Psychiatry and the Behavioral Sciences, Keck School of Medicine of University of Southern California, Los Angeles, CA, United States; ^2^Mailman School of Public Health, Columbia University, New York, NY, United States; ^3^Columbia University, New York, NY, United States; ^4^Department of Medicine-Pediatrics, David Geffen School of Medicine, Los Angeles, CA, United States

**Keywords:** discrimination, insurance, insurer, LGBTQ+, payer, stigma, trust

## Abstract

**Introduction:**

LGBTQ+ individuals have historically faced and continue to experience stigma and discrimination in various areas, including healthcare. There is very limited data regarding LGBTQ+ people’s perceptions of their health insurer and health insurer workers.

**Methods:**

An online cross-sectional survey was conducted with a national sample of United States residents, who responded to questions about their healthcare, including experiences with their health insurer and health insurer workers.

**Results:**

Compared to cisgender, heterosexual people (*n* = 1,400), LGBTQ+ people (*n* = 1,234) reported significantly poorer experiences with their health insurer, including being dissatisfied with their health insurer; believing their health insurer is not their advocate; distrusting their health insurer; not knowing what is covered in their health plan; being dissatisfied with providers in their health plan; and not believing their health insurer meets their needs. Additionally, compared to cisgender, heterosexual people, LGBTQ+ people conveyed poorer experiences with health insurer workers, including health insurer workers not addressing them by their names; not being comfortable when interacting with them; not being coordinated; misgendering them; and being discriminatory toward them.

**Discussion:**

LGBTQ+ communities continue to face significant healthcare disparities, including stigma and discrimination from health insurers and health insurer workers. Longitudinal dedication to LGBTQ+ education, advocacy, and institutional reform is necessary to dismantle the entrenched discrimination in health insurer environments and create more equitable, supportive environments for all LGBTQ+ people.

## Introduction

In 2010, the Affordable Care Act (ACA) was signed into law with the goal of making affordable health insurance available to more individuals in the United States (U. S.). The Affordable Care Act also included a non-discrimination provision (Section 1557) that prohibits discrimination on the basis of race, color, national origin, age, disability (including human immunodeficiency virus (HIV) status), or sex in any federally supported health programs or activities (National Academies of Sciences, Engineering, and Medicine, 2020). This enactment established that health insurance companies were legally barred from denying, canceling, or refusing to issue or renew plans or policies; limiting coverage of claims; imposing additional cost sharing or additional limitations or restrictions on coverage based on one’s demographics; or implementing discriminatory marketing practices (Rosenbaum, 2016). Before the implementation of the ACA and Section 1557, discrimination in the health insurance market was palpable and existed in various forms. Insurers often had exclusionary policies through eligibility discrimination whereby insurers used an individual’s health status to determine whether that person could enroll in a plan or to dictate that person’s monthly premium (Guo et al., 2017). Additional forms of discrimination included adverse tiering, when insurers would position certain drugs, such as HIV antiretrovirals, in high cost-sharing levels (Jacobs and Sommers, 2015).

After the ACA and the landmark Supreme Court decision under Obergefell v. Hodges, studies have shown that there has been progress in the number of lesbian, gay, bisexual, transgender, queer, and all sexually diverse and gender diverse (LGBTQ+) individuals who are insured. One study indicated that uninsurance declined by 5% for nonelderly sexually diverse adults between 2013–2014 and 2017–2018 (Gonzales et al., 2021). A similar study conducted between 2013–2019 found that by the end of the study period, there was no statistically significant difference in overall insurance coverage rates for LGBTQ+ adults and cisgender, heterosexual adults (Bolibol et al., 2023).

However, despite great strides in insured rates amongst LGBTQ+ individuals, there is still little research that documents the experiences of LGBTQ+ consumers with their insurers post-ACA. Insurance companies play unique roles in healthcare (Sandhu et al., 2024). Some perspectives have likened insurers to “gatekeepers” as they mediate patient rights and provide coverage for procedures, drugs, and services that they deem “medically necessary” (Kirkland et al., 2021). Insurers often use aggregations of medical diagnoses, established standards of care, value judgments, and business calculations to determine whether specific treatments can be authorized and covered (Kirkland et al., 2021). Additionally, coverage for treatment, services, and procedures related to gender-affirming care varies widely across insurers and U.S. states (National Academies of Sciences, Engineering, and Medicine, 2020; Sandhu et al., 2024). Therefore, despite past progressive legal reforms, there is still prominent ambiguity regarding what type of care constitutes as medically necessary. This reality can lead to LGBTQ+ individuals navigating complicated, perplexing terrain which may lead to distrust and dissatisfaction with their insurance companies (Nowaskie et al., 2025).

In general, LGBTQ+ data is disproportionately underrepresented in research. Research has consistently shown that compared to cisgender, heterosexual people, LGBTQ+ individuals face substantial stigmas within healthcare which may lead to fear of services or avoidance of healthcare altogether (Sileo et al., 2022), thereby exacerbating chronic physical and mental health conditions. While there is some data on prevalent health outcome disparities among LGBTQ+ communities, there is much less devotion to examining LGBTQ+ healthcare experiences (Nowaskie et al., 2025). Although a scant amount explores LGBTQ+ narratives of healthcare provider and staff interactions, there is no known published data regarding LGBTQ+ perspectives about healthcare insurance companies (“health insurers”) and health insurer workers. As such, this research aimed to investigate LGBTQ+ people’s healthcare experiences with their insurance companies in comparison to cisgender, heterosexual people’s experiences.

## Materials and methods

Between March to April 2022, an anonymous, self-report, cross-sectional survey was distributed online via a third-party vendor to a national sample of United States (U. S.) residents. Participation was voluntary and constituted consent. Participants self-disclosed their age, sexual orientation, gender identity, race/ethnicity, education, employment, individual annual income, and region of residence. Participants also answered questions about their healthcare experiences. This data was provided by Optum, who commissioned the survey, to a co-author. Because data was deidentified, this study was deemed not human subjects research by the University of Southern California Institutional Review Board (Protocol #UP-24-00659).

Participants were weighted to be nationally representative of the general U. S. population based on demographic variables. Participants were then categorized into two groups based on their sexual orientation and gender identity: (1) LGBTQ+ and (2) exclusively cisgender and heterosexual; some participants (*n* = 79) were excluded from analyses as they had preferred not to disclose either their sexual orientation and/or gender identity. Similarly, given the study’s focus to understand healthcare experiences with insurance companies, some participants (*n* = 268) were excluded from analyses as they did not have insurance.

Frequencies of demographic and healthcare insurer experience questions were computed. Differences between LGBTQ+ people and cisgender, heterosexual people were calculated using chi-square tests. Multivariate linear regression models were conducted with health insurer experiences (1 = directional favorability, 3 = directional unfavorability) as dependent variables and age, education (1 = more than 4-year college degree, 6 = 8th grade or less), employment (1 = working full-time, 4 = unemployed), identity (0 = cisgender, heterosexual, 1 = LGBTQ+), individual annual income (1 = more than $150,000, 5 = less than $20,000), insurance type (1 = employer-based, 4 = self-pay), and race/ethnicity (1 = White or Caucasian, 10 = multiple identities) as covariates. Given the survey length, question magnitude, and focus of this manuscript, specific items related to perceptions of health insurers are reported here.

Additionally, it is well known that many demographic variables, including age, race/ethnicity, education, employment, individual annual income, and state of residence, may influence health outcomes. To avoid difficult interpretations due to analytical complexity, comparative analyses reported here are based solely on sexual orientation and gender identity, i.e., LGBTQ+ people compared to cisgender, heterosexual people.

## Results

After weighting, a total of 2,713 people expressed their perspectives and voices, including LGBTQ+ people (*n* = 1,313) and cisgender, heterosexual people (*n* = 1,400) (Table 1). Majority of LGBTQ+ people were bisexual, cisgender, between 20 to 50 years old, White/Caucasian, had higher education, employed, and earned more than $20,000 annually; they lived across the U. S.

**Table 1:** Demographics across LGBTQ+ and cisgender, heterosexual samples.

	LGBTQ+ people (*n* = 1,313)	Cisgender, heterosexual people (*n* = 1,400)
Age	34.93 (14.58)	47.52 (16.44)
Sexual orientation		
Asexual	38 (2.9%)	
Bisexual	814 (62.0%)	
Gay	180 (13.7%)	
Heterosexual/straight	80 (6.1%)	1,400 (100.0%)
Lesbian	164 (12.5%)	
Pansexual	64 (4.9%)	
Queer	81 (6.2%)	
Additional identities	13 (1.0%)	
Prefer not to disclose	8 (0.6%)	
Gender identity		
Agender	86 (6.5%)	
Cisgender man	245 (18.7%)	700 (50.0%)
Cisgender women	580 (44.2%)	700 (50.0%)
Genderqueer	37 (2.8%)	
Nonbinary	188 (14.3%)	
Transgender man	73 (5.6%)	
Transgender woman	48 (3.7%)	
Additional identities	62 (4.7%)	
Prefer not to disclose	71 (5.4%)	
Race/ethnicity		
Alaska Native	2 (0.2%)	0 (0%)
American Indian	13 (1.1%)	12 (0.9%)
Asian or Asian American	47 (3.9%)	73 (5.2%)
Black or African American	187 (15.3%)	123 (8.8%)
Hispanic and/or Latino/a/x	101 (8.3%)	78 (5.6%)
Native Hawaiian	3 (0.2%)	1 (0.1%)
Pacific Islander	6 (0.5%)	1 (0.1%)
White/Caucasian	717 (58.8%)	1,049 (75.4%)
Combinations	130 (10.7%)	43 (3.1%)
Additional identities	14 (1.1%)	12 (0.9%)
Education		
8th grade or less	5 (0.4%)	2 (0.1%)
Some high school but did not graduate	54 (4.4%)	34 (2.4%)
High school graduate or GED	383 (31.3%)	339 (24.3%)
Some college or 2-year degree	463 (37.8%)	445 (31.9%)
4-year college graduate	216 (17.6%)	372 (26.7%)
More than 4-year college degree	103 (8.4%)	203 (14.6%)
Employment		
Unemployed	296 (27.2%)	198 (15.1%)
Working part-time	246 (22.6%)	171 (13.1%)
Working full-time	439 (40.4%)	608 (46.4%)
Retired	106 (9.8%)	333 (25.4%)
Individual annual income		
Less than $20,000	419 (35.8%)	264 (19.6%)
$20,001–$50,000	415 (35.4%)	429 (31.9%)
$50,001–$100,000	239 (20.4%)	420 (31.2%)
$100,001–$150,000	73 (6.2%)	148 (11.0%)
More than $150,000	26 (2.2%)	84 (6.2%)
Region of residence		
Midwest	238 (19.3%)	339 (24.2%)
Northeast	247 (20.0%)	289 (20.6%)
South	471 (38.2%)	504 (36.0%)
West	277 (22.5%)	268 (19.1%)
Insurance type		
Employer-based	329 (27.7%)	568 (41.2%)
Government-based	651 (54.9%)	665 (48.2%)
Additional types	53 (4.5%)	32 (2.3%)
None/self-pay	153 (12.9%)	115 (8.3%)

Overall, significantly more LGBTQ+ people reported poorer experiences with their health insurer than cisgender, heterosexual people (Table 2), such as (1) being dissatisfied with their health insurer, (2) believing their health insurer is not their advocate, (3) distrusting their health insurer, (4) not knowing what is covered in their health plan, (5) being dissatisfied with providers in their health plan, and (6) not believing their health insurer meets their needs. Additionally, compared to cisgender, heterosexual people, LGBTQ+ people conveyed poorer experiences with health insurer workers, such as health insurer workers (1) not addressing them by their names, (2) not being comfortable when interacting with them, (3) not being coordinated, (4) misgendering them, and (5) being discriminatory toward them. In the multivariate linear regression models, LGBTQ+ identity was a statistically significant predictor for many of the health insurer experiences, including (1) being dissatisfied with their health insurer, (2) believing their health insurer is not their advocate, (3) distrusting their health insurer, (4) being dissatisfied with providers in their health plan, (5) not believing their health insurer meets their needs, and (6) health insurer workers not being coordinated.

**Table 2:** Perceptions of health insurers across LGBTQ+ and cisgender, heterosexual samples.

	Disagree/Strongly disagree		Neither agree nor disagree		Agree/Strongly agree		$\chi^{2}(2)$	Model fit *F*(23, 1,263)	$n_{p}^{2}$
	LGBTQ+	Cisgender, heterosexual	LGBTQ+	Cisgender, heterosexual	LGBTQ+	Cisgender, heterosexual			
I am satisfied with my health insurer.	139 (13.8%)	90 (7.2%)	232 (23.0%)	215 (17.3%)	636 (63.2%)	938 (75.5%)	44.815^***^	4.689***	0.013***
My health insurance plan is affordable.	136 (13.9%)	141 (11.5%)	219 (22.4%)	257 (21.0%)	623 (63.7%)	828 (67.5%)	4.235	3.312***	0.001
My health insurer is an advocate for me.	182 (18.7%)	143 (12.0%)	339 (34.8%)	390 (32.7%)	453 (46.5%)	660 (55.3%)	24.868^***^	3.249***	0.007**
I trust my health insurer.	159 (15.9%)	100 (8.1%)	250 (25.1%)	258 (20.8%)	589 (59.0%)	883 (71.2%)	46.461^***^	3.436***	0.009***
I know what is covered by my health insurance plan.	196 (19.5%)	129 (10.4%)	199 (19.8%)	244 (19.6%)	612 (60.8%)	869 (70.0%)	38.850^***^	3.800***	0.003
I am satisfied with the network of health care providers included in my plan.	126 (12.6%)	86 (7.0%)	229 (22.9%)	211 (17.1%)	646 (64.5%)	939 (76.0%)	38.181^***^	3.500***	0.004*
My health insurer benefits meet my needs.	154 (15.3%)	88 (7.1%)	205 (20.4%)	212 (17.1%)	647 (64.3%)	942 (75.8%)	48.645^***^	3.812***	0.007**
Health insurer workers have addressed me by the name I go by.	68 (8.8%)	43 (4.6%)	101 (13.1%)	109 (11.6%)	603 (78.1%)	791 (83.9%)	14.383^***^	2.617***	0.000
Health insurer workers have been comfortable when interacting with me.	47 (6.0%)	31 (3.2%)	145 (18.6%)	115 (12.0%)	586 (75.3%)	814 (84.8%)	25.091^***^	2.593***	0.003
Health insurer workers have been coordinated.	101 (13.0%)	53 (5.6%)	179 (23.0%)	208 (21.9%)	497 (64.0%)	688 (72.5%)	31.088^***^	2.109**	0.003*
I plan to renew my coverage with my health care insurer.	86 (8.8%)	79 (6.5%)	202 (20.7%)	222 (18.4%)	690 (70.6%)	906 (75.1%)	6.545^*^	2.781***	0.000
Health insurer workers have misgendered me.	150 (20.4%)	118 (13.5%)	91 (12.4%)	74 (8.5%)	494 (67.2%)	681 (78.0%)	23.664^***^	6.001***	0.000
Health insurer workers have been discriminatory.	141 (18.3%)	129 (13.9%)	115 (15.0%)	123 (13.2%)	513 (66.7%)	679 (72.9%)	8.560^*^	4.323***	0.000

Models were controlled for age, race/ethnicity, education, employment, individual annual income, insurance type, and identity. Reported $n_{p}^{2}$ are for effect sizes of the identity variable. *p < 0.01; **p < 0.01; ***p < 0.001.

### Discussion

The purpose of this study was to investigate LGBTQ+ individuals’ perceptions with their health insurers. Overall, data revealed that compared to cisgender, heterosexual people, LGBTQ+ people reported significantly poorer experiences with their health insurer companies and poorer experiences with health insurer workers. These challenges included dissatisfaction, distrust, and confusion about healthcare coverage as well as negative interactions with health insurer workers, such as misgendering, discomfort during interactions, and similar discriminatory behaviors. These findings highlight how health insurers, often acting as gatekeepers as described by Kirkland et al. (2021), may erode trust among patients through opaque policies and discretionary practices that disproportionately impact LGBTQ+ populations.

The 2010 Affordable Care Act (ACA), alongside Section 1557’s non-discrimination provisions, was enacted with the goal of reducing discrimination in healthcare by prohibiting discriminatory practices, such as denying coverage based on demographics or health status (National Academies of Sciences, Engineering, and Medicine, 2020). Studies have demonstrated that since the ACA, insurance coverage rates particularly among LGBTQ+ individuals have increased (Bolibol et al., 2023; Gonzales et al., 2021). However, this study demonstrates that while these legal reforms marked progress in insurance access and maintenance, they did not completely eliminate stigma and discrimination. For example, LGBTQ+ individuals still face substantial barriers related to ambiguous coverage for gender-affirming care and gatekeeping practices by health insurers that perpetuate distrust (Kirkland et al., 2021; National Academies of Sciences, Engineering, and Medicine, 2020; Sandhu et al., 2024). More recently, the 2025 U.S. federal administrated declared designation of only two sexes (“male” and “female”) and insinuated that sex is unchangeable; additionally, the administration largely disregarded recognition of gender identity and gender ideology (The White House, 2025). Over time, this executive order will likely lead to and exacerbate significant sociopolitical marginalizations, shifts in insurance policies and coverages, and subsequent healthcare stigmas and health outcome disparities, especially for gender diverse people. These past findings and recent policy changes indicate that much more work is needed to address discriminatory practices within health insurer cultures and ultimately dismantle institutional and systemic discriminations.

The lack of awareness and attention to LGBTQ+ care and equity within health insurer environments and subsequent lack of knowledge, preparedness, and affirmation among health insurer workers can certainly exacerbate negative experiences and poor health outcomes. Past research has consistently shown that healthcare providers, staff, and systems have limited education and training in LGBTQ+ topics and exposure to LGBTQ+ people, which in turn leads to pervasive biases and stigmas toward LGBTQ+ communities (Nowaskie and Garrison, 2024; Safer et al., 2016). Over time, this longstanding overt disregard has consequently intensified LGBTQ+ people distrusting healthcare staff and systems; delaying, avoiding, and foregoing routine and preventive care; and sometimes seeking unregulated, unsafe treatments and interventions (Nowaskie et al., 2025; Sileo et al., 2022). These vicious cycles of marginalization often worsen physical and mental health conditions, overall well-being, and quality of life (Nowaskie and Garrison, 2024; Safer et al., 2016). Similarly, health insurers are positioned in parallel roles of care, often potentiating barriers to care with insufficient access to LGBTQ+ affirming providers and services (Sandhu et al., 2024).

Despite some advancement in legal protections for LGBTQ+ individuals, these data highlight that significant efforts are still required to alleviate current and prevent future stigma and discrimination within health insurance industries. A critical area for improvement involves increasing education, training, and exposure to LGBTQ+ care and equity topics with health insurer workplace environments (Nowaskie and Garrison, 2024). Additional necessities include instituting affirming policies and increasing coverage for services, especially gender affirming care (Sandhu et al., 2024). Clearly, many health insurers and health insurer workers are not effectively nor affirmingly engaging with nor supporting LGBTQ+ people. These systemic barriers require longitudinal commitments and partnerships to change. National nonprofit organizations such as OutCare Health provide valuable information, resources, education, programming, and advocacy to promote affirming practices in healthcare settings across entire institutional ecosystems (Nowaskie, 2021; Nowaskie and Garrison, 2024). Expanding these efforts, alongside implementing systemic reforms to address discriminatory practices, is essential for fostering more equitable healthcare landscapes.

## Limitations

Several key limitations were apparent in this study. There may have been underreported or overreported experiences as well as inherent biases within survey participants. Although data was weighted to be a representative national U. S. sample, the survey’s online format may have limited generalizability, e.g., not fully representing people living in rural areas, people with low socioeconomic status, people who are less comfortable with technology, and people without internet access. While the survey collected a broad range of demographic data, it did not specifically consider nor directly address intersectionality across various LGBTQ+ subgroups. This approach limits the study to a more homogenized understanding of LGBTQ+ peoples’ experiences rather than a collection of diverse subgroups and communities across multiple intersecting identities. With the amount of collected data, multiple analyses could have been undertaken, incorporating variables such as age, race/ethnicity, education, employment, income, region, and distinct sexual orientations and gender identities. More in-depth explorations are crucial, especially comparing experiences across specific LGBTQ+ subgroups (such as gender diverse individuals and LGBTQ+ people of color) to better understand the nuances in healthcare insurer experiences and the disparities that may exist among these particular subgroups.

## Conclusion

LGBTQ+ communities continue to face significant healthcare disparities, including stigma and discrimination from health insurers and health insurer workers. Specifically, LGBTQ+ people have poor experiences with health insurers (including dissatisfaction and distrust) and negative interactions with health insurer workers (such as misgendering and discomfort). These stigmas and discriminations perpetuate pervasive barriers to care and equity for LGBTQ+ communities. To address these systemic disparities, it is crucial to implement comprehensive training, institute inclusive policies, and ensure full coverage of services. Longitudinal dedication to LGBTQ+ education, advocacy, and institutional reform is necessary to dismantle the entrenched discrimination in health insurer environments and create more equitable, supportive environments for all LGBTQ+ people.

## Data Availability

The original contributions presented in the study are included in the article/supplementary material, further inquiries can be directed to the corresponding author/s.
